# Combined Treatment of a Gallbladder Volvulus with a Common Bile Duct Obstruction

**DOI:** 10.1155/2017/3906042

**Published:** 2017-04-30

**Authors:** M. Laydi, K. Charpentier, B. Paquette, B. Heyd

**Affiliations:** Surgery Department, CHU Besançon, 25000 Besançon, France

## Abstract

Gallbladder volvulus is a rare disease and can lead to an acute cholecystitis. We report the case of an elderly woman with a gallbladder volvulus, diagnosed at CT scan and treated by surgery and endoscopic sphincterotomy.

## 1. Introduction

Gallbladder volvulus is defined by a gallbladder rotation around its mesentery along the axis of the cystic duct and artery [1]. Torsion occurs when “free-floating” gallbladder twists around its pedicle, leading to an obstruction of biliary drainage and blood flow [2]. This disease course is gangrenous gallbladder and biliary peritonitis. Initially described by Wendel in 1898 [3], the gallbladder volvulus is a relatively rare entity [4]. With approximately 500 cases described in literature [5] most of them were reported in elderly people with a ratio female-to-male of 3 : 1 [6].

## 2. Case Report

**Figure 1:**
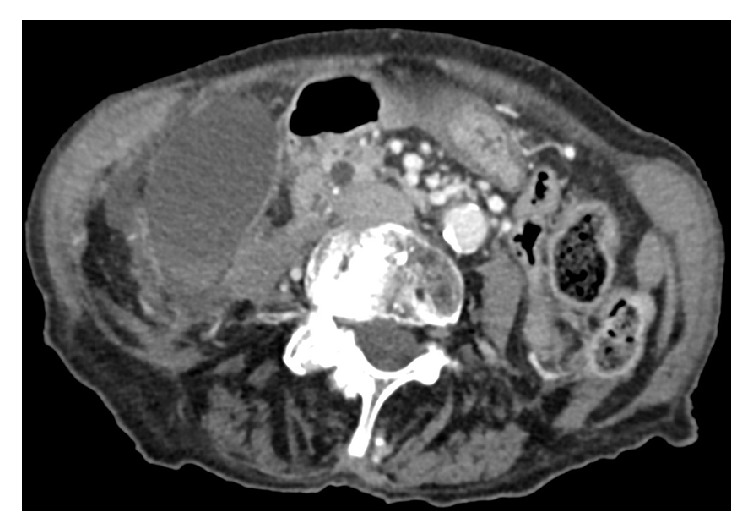
Abdominal contrast enhanced CT scan, portal phase distended gallbladder with focal enhancement defect.

**Figure 2:**
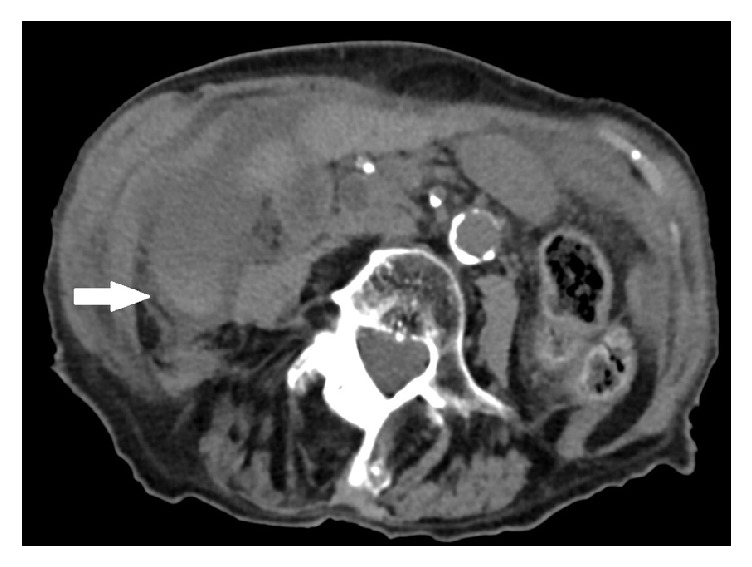
Abdominal unenhanced CT scan: high attenuation area, suggesting an intraluminal hemorrhage.

**Figure 3:**
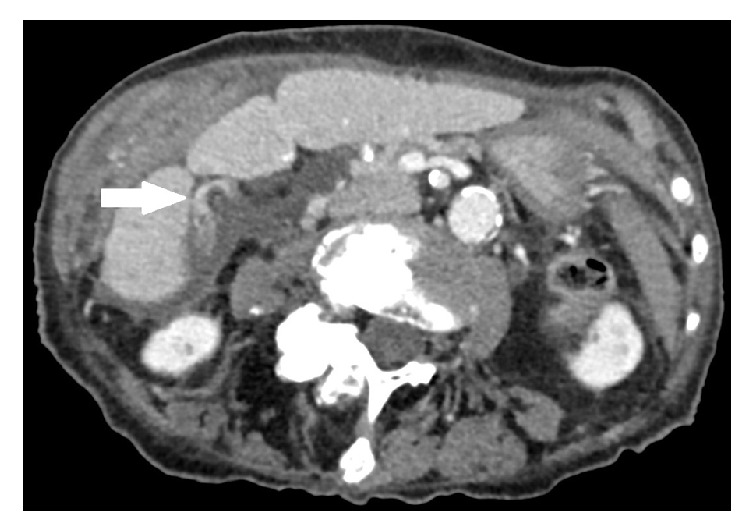
Abdominal contrast enhanced CT scan, portal phase: “Whirl sign” of the gallbladder pedicle.

**Figure 4:**
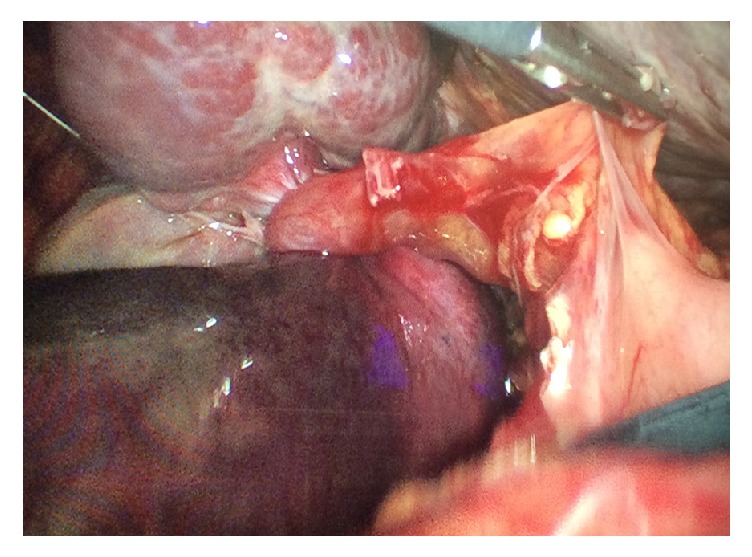
Laparoscopic view.

An 89-year-old woman was admitted to our hospital. Her reason for admission was an acute pain from the right hypochondriac region and constipation. The patient's past medical history included a severe hypertensive and valvular heart disease, chronic kidney failure, right carotid stenosis, hiatal hernia, colon angiodysplasia, and a Raynaud's syndrome. Her surgical history included rectocele cure and appendectomy. The spasmodic pain was localized at the right hypochondriac region since less than 24 hours. At admission her temperature was 36.3°C, her pulse was 58 bpm, and blood pressure was 146/69 mmHg. Her BMI was 19 kg/m^2^. Physical examination showed right hypochondriac Murphy's sign with defense but no contracture. There was no jaundice. The patient was not nauseous. Biology showed a leukocyte count of 14.2 × 10^9^/L (normal, 4–10 × 10^9^/L) a C reactive protein of 25.6 mg/L (normal, <8 mg/L). Full liver function test was normal. Serum lipase level was normal. She had a hyponatremia of 127 mmol/L (normal, 135–145 mmol/L) and an normocytic anemia of 10.9 g/dL (normal, 12–16 g/dL). A CT scan with was realized with contrast injection. Cholecystitis was revealed by a gallbladder enlargement (Figure 1) with intraluminal hemorrhage (Figure 2), wall thickening, and free fluid. No stones were detected. Focal enhancement defects (Figure 1) suggested ischemic phenomena and preperforative gallbladder. Torsion was revealed by a whirl sign [7] of the cystic pedicle (Figure 3) and lateral position of the gallbladder neck. This abnormal configuration is one of the most evocative signs of gallbladder volvulus. An emergency laparoscopic cholecystectomy with a rendezvous technique was performed. Surgery revealed a necrotic gallbladder, rotated to 720 degrees clockwise around its mesentery (Figure 4). Detorsion and cholecystectomy were performed with success. Peroperative cholangiography showed gallstones impacted in the proximal part of the bile duct. Duct cannulation and sphincterotomy by electrosurgical division of papilla was done. The 1 cm gallstone was retrieved with a balloon sweep and the procedure was finished by the set-up of a suction drainage. Pathological examination revealed a 9,5 cm necroinflammatory and hemorrhagic gallbladder. There was no sign of malignancy and it was stone free. Global cardiac failure from an acute coronary syndrome worsened during her postoperative course; medical management could not avoid her death on postoperative day 12.

## 3. Conclusion

Sudden cholecystitis symptoms, particularly in elderly patient with a few risk factors should always evoke a gallbladder volvulus. The prompt diagnosis is crucial to ensure that the patient undergoes an emergent cholecystectomy rather than temporizing measures with antibiotics. Risks factors and pathology results should help lead to this diagnostic. The patient medical history showed many contributing factors [8] as an iatrogenic manipulation of the abdomen (She had a coloscopy 8 weeks before), colon angiodysplasia, malnutrition, and hiatal hernia. Other risks factors [8] are anatomic variants like an unusually long mesentery, hypermobile liver, or kyphoscoliosis. Gallstones are not a risk factor, only reported from 20 to 33% of cases [9]. However diagnosis remains difficult and no imaging mean has proven to be sufficiently sensitive [9].
